# Epicardial pacing lead implantation for congenital complete atrioventricular block immediately after birth: a case report

**DOI:** 10.1186/s13256-023-04190-8

**Published:** 2023-11-01

**Authors:** Hiroaki Amino, Mao Kinoshita, Masayuki Shibasaki

**Affiliations:** Department of Anesthesiology, Kyoto Prefectural University of Medicine, Kyoto, 602-8566 Japan

**Keywords:** Congenital complete atrioventricular block, Internal ventricular pacing lead implantation, Seamlessly

## Abstract

**Background:**

The incidence of congenital complete atrioventricular block is estimated to be 1 per 20,000 deliveries. In the fetal period, the fetal mortality rate is high, but the treatment strategy has not yet been established. In severe cases, early postnatal pacing therapy is necessary.

**Case presentation:**

A 0-day-old Japanese baby girl was diagnosed with fetal congenital complete atrioventricular block during a prenatal physical examination. A joint conference was held preoperatively among multidisciplinary departments, and a cesarean section was performed at 37 weeks pregnancy, immediately followed by scheduled internal ventricular pacing lead implantation in an adjacent room. Percutaneous pacing was ineffective. The epicardial pacing lead was sutured at 17.5 minutes after birth, and perioperative management was successful with a heart rate and pulse rate of 150 beats per minute.

**Conclusion:**

The infant with a congenital complete atrioventricular block was rescued by an uneventful epicardial lead implantation.

## Introduction

The frequency of congenital complete atrioventricular block (CAVB) is estimated to be 1 case per 20,000 deliveries [1]. The causes of CAVB are broadly classified into nonimmunological and immunological. Immunological causes include anti-Sicca syndrome A/Ro (SS-A/Ro) and Sicca syndrome B/La (SS-B/La) antibodies [2]. These pass through the placental circulation and cause immune-mediated inflammation or fibrosis in the fetal conduction heart tissue, which may cause conversion [3, 4]. In addition, predictors of poor prognosis include a heart rate (HR) of less than 50 beats per minute, diagnosis at less than 20 weeks of gestation, fetal edema, and left ventricular dysfunction. In the presence of these risk factors, fetal and neonatal mortality rates are as high as 22% and 18%, respectively [5].

In severe cases, pacemaker implantation early in the neonatal period is considered necessary; however, there are many challenges including small-sized hearts that make implantation difficult, battery replacement and mode changes in the remote period, and development of dilated cardiomyopathy. We thought it important to share our clinical experience on this rare topic of congenital pathology by presenting a report of a case wherein life-saving epicardial pacing leads were placed immediately after a cesarean section. Written informed consent was obtained from the patient's parents for publication of this case report. This manuscript adheres to the applicable CARE guideline.

## Case presentation

**Fig. 1:**
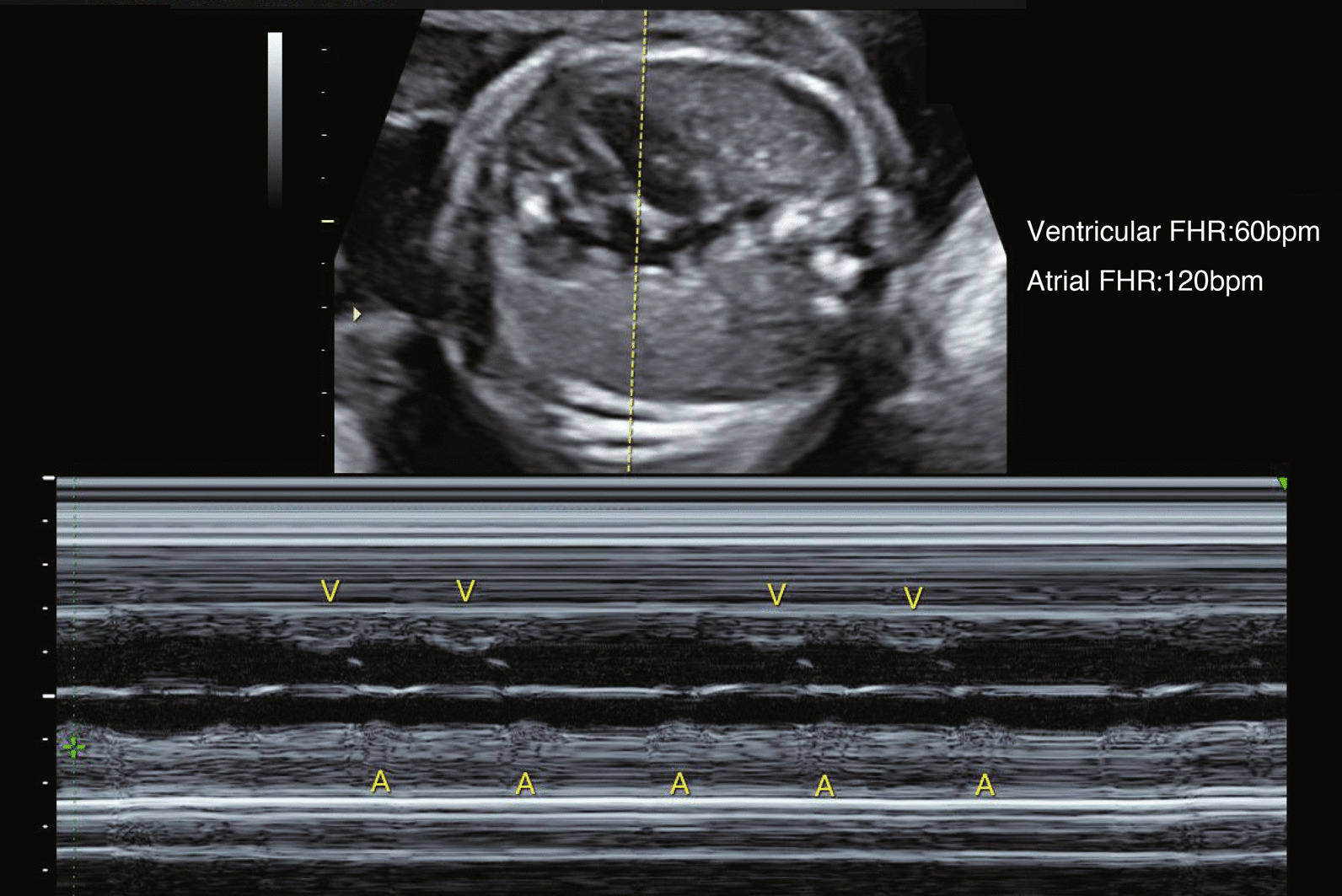
M-mode fetal ultrasound scan. Complete atrioventricular block is apparent by the atrial and ventricular wall movement recordings. *A* atrial contraction, *V* ventricular contraction, *FHR* fetal heart rate

**Fig. 2:**
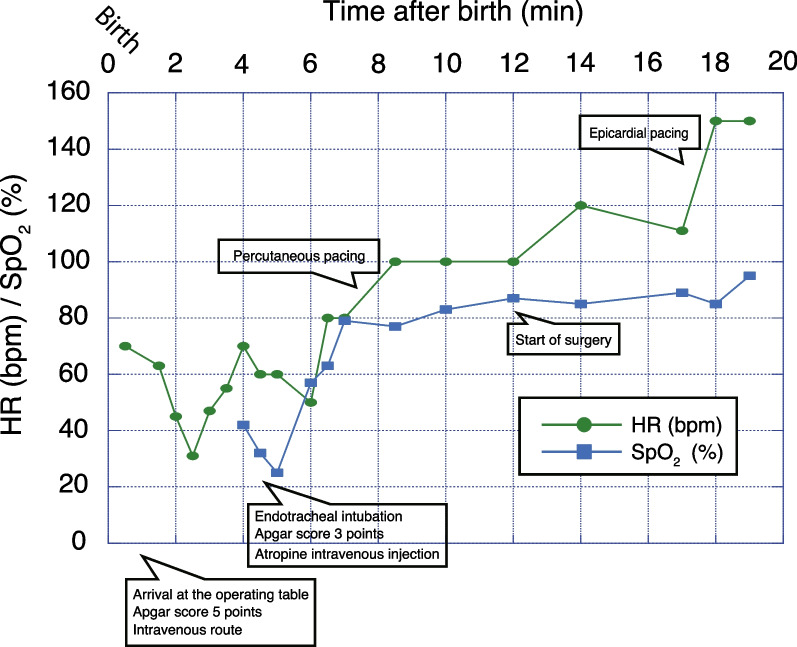
Anesthesia record. Although percutaneous pacing was started, the HR did not improve and the pulse rate was half of the pacing settings (time after birth). *HR* heart rate, *bpm* beats per minute, *SpO*_*2*_ peripheral blood oxygen saturation

Our case is of a 0-day-old Japanese baby girl. Fetal bradyarrhythmia was noted during a prenatal checkup of the mother at 23 weeks 3 days of gestation. A fetal ultrasound scan revealed fetal rhythm abnormality, with a fetal heart rate (HR) of 50–60 beats per minute and a high degree of bradycardia (Fig. 1). Thus, maternal beta-stimulants and steroids were started. The mother was admitted to the hospital at 36 weeks 1 day of gestation for maternal and fetal management. Preoperative examination revealed that the mother was positive for SS-A/Ro antibodies (3000 U/mL) and negative for SS-B/La antibodies; thus, immunological mechanisms were assumed to be the cause of the cardiac abnormalities. A fetal ultrasound examination at 36 weeks 5 days of gestation revealed the following: estimated fetal weight of 1995 g, cardiothoracic area ratio of 34%, atrial rate of 136 beats per minute, ventricular rate of 66 beats per minute, moderate pulmonary atresia, and short-circuited right and left ductus arteriosus with patency. After a joint preoperative conference with the departments of anesthesiology, pediatric cardiovascular surgery, obstetrics and gynecology, pediatrics, and the operating room nurses and midwives, a cesarean section was performed under spinal subarachnoid and epidural anesthesia at 37 weeks 1 day of gestation. The delivery room was staffed by an anesthesiologist, pediatrics, obstetrics and gynecology, and nurses. The infant was scheduled for immediate epicardial pacing lead placement in the adjacent room. The first goal was to start pacing in the shortest possible time, then to secure rooms in the operating theaters next to each other. The fetus was carefully kept warm by wiping the fetal sebum thoroughly. Intravenous lines were inserted, and if difficult to secure, an umbilical catheter was placed. Percutaneous pacing was performed, but priority was given to disinfection and surgical procedures. If the right ventricle was difficult to secure owing to poor threshold in epicardial pacing, transvenous pacing would be performed. *Ex utero* intrapartum treatment was not available at our facility owing to the lack of experience to perform it. The infant was scheduled for immediate epicardial pacing lead placement in the adjacent room. She weighed 1764 g, cried, and had muscle tone; however, the fetal HR decreased to 40 beats per minute and generalized cyanosis appeared; bag-mask ventilation was started immediately. Subsequently, tracheal intubation was performed by a neonatologist, and sevoflurane, rocuronium, fentanyl, and atropine were administered before the surgery began. Although percutaneous pacing was started, the HR did not improve and the pulse rate was half of the pacing settings. There was no response to atropine, and isoproterenol was prepared but not used because the respiratory–circulatory dynamic (though relatively unstable) was maintained. The epicardial pacing lead was attached, and the setting was adjusted to the DDD mode (170 beats per minute, atrial output 2.0 mA, and ventricular output 2.0 mA). The operation time was 89 minutes, and the anesthesia time was 127 minutes. Figure 2 shows the anesthesia record until the epicardial pacing lead was attached. The pacing threshold gradually increased after the operation, and a permanent pacemaker implantation was performed on day 72; the patient was discharged on day 91.

## Discussion

Only a few cases of perioperative anesthesia management of congenital CAVB, with epicardial pacing leads implanted a few hours after cesarean section, have been reported [6]; however, in these few cases, implantation was performed immediately after birth. In the present case, perioperative management was performed for a time-constrained fetus immediately after birth, and epicardial pacing implantation was performed quickly and safely due to the ineffectiveness of drug therapy or percutaneous extracorporeal pacing.

Perioperative anesthesia management of congenital CAVB with implantation of epicardial pacing leads immediately after cesarean section is very rare. No previous reports have presented the optimal timing of postnatal pacing lead implantation; the timing has varied from the day of birth to several days after birth [7, 8]. In this case, we planned to perform intraventricular pacing lead placement in an adjacent room immediately after a scheduled cesarean section at 37 weeks 1 day of gestation, considering that pacing lead placement is necessary after birth and that the timing of the placement should be as early as possible. To ensure a smooth procedure, joint conferences were held several times among the departments of anesthesiology, pediatric cardiovascular surgery, obstetrics and gynecology, pediatrics, and the operating room nurses and midwives beforehand to carefully confirm the surgical procedure and preparations. In this case, the postnatal HR was highly depressed at 40 beats per minute and cyanosis occurred that could be treated by prompt placement of a pacing lead in the infant. Issues to be addressed include replacing the pacemaker battery in the remote period, changing the pacing mode as the child grows, poor pacing, and transitioning to dilated cardiomyopathy in the future.

Perioperative management of a time-constrained postnatal fetus immediately allowed for the safe implementation of epicardial pacing implantation in the face of ineffective pharmacological therapy and percutaneous extracorporeal pacing. Isoproterenol was prepared but not used because the operation was started promptly and the respiratory–circulatory dynamic was relatively stable; however, we should have considered esophageal pacing. Treatment strategies for congenital CAVB have not yet been established. Reported *in utero* management includes the use of beta-stimulants, anti-inflammatory therapies such as steroids, and intravenous immunoglobulin (IVIG). Beta-stimulants have been reported to increase the fetal ventricular rate, but studies have not demonstrated an increase in fetal HR and have not shown a survival advantage [2]. Steroids have been reported to be effective in treating fetal heart failure, but have not been shown to improve prognosis; issues that must be addressed include their safety and side effects in the mother and the child [2]. IVIG aims to minimize fetal heart injury by reducing autoantibodies in the maternal blood; however, again, no studies have shown clear efficacy [5]. Regarding postnatal treatment strategies, it is recommended to perform a cesarean delivery and wait until the infant has gained sufficient weight before pacemaker implantation [7, 9]. If the infant is small, it is recommended to wait for lung maturation under ventilatory management, beta-stimulant administration, and pacing lead implantation to prevent heart failure [7, 9]. However, the fetal and neonatal mortality rates are high, and immediate pacing and heart failure control are considered necessary in severe cases with one or more of the following poor prognostic factors: HR less than 50 beats per minute, diagnosis at less than 20 weeks of gestation, fetal edema, and left ventricular dysfunction. Percutaneous fetal implantation of cardiac pacemakers has been shown to be effective in experimental studies, suggesting that it may be applied in edematous human fetuses with CAVB; however, no studies have demonstrated its efficacy in human fetuses [4, 10]. In the present case, the fetal HR was less than 55 beats per minute and mild fetal edema was observed; therefore, we considered the patient to be severely ill and managed her perinatally.

The fetus of a pregnant woman who is positive for autoantibodies should be monitored by fetal echocardiography, and the timing of pacemaker implantation should be considered. In addition, not all facilities have the manpower and environment to respond to cases such as this one, so it is necessary to establish a method to identify cases, such as this one, that should be handled promptly.

## Conclusion

Congenital CAVB is a rare disorder, but mortality is high in severe cases. However, treatment strategies have not yet been established. In this study, we report the case of a child with congenital CAVB who was successfully treated immediately after birth with epicardial pacing lead implantation and survived.

## Data Availability

Not applicable.

## Abbreviations

CAVB: Complete atrioventricular block
HR: Heart rate
IVIG: Intravenous immunoglobulin
